# Precise treatment of aortic aneurysm by three-dimensional printing and simulation before endovascular intervention

**DOI:** 10.1038/s41598-017-00644-4

**Published:** 2017-04-11

**Authors:** Ding Yuan, Han Luo, Hongliu Yang, Bin Huang, Jingqiang Zhu, Jichun Zhao

**Affiliations:** 1 Department of Thyroid & Parathyroid Surgery, West China Hospital, Chengdu, P.R. China; 2 Department of Vascular Surgery, West China Hospital, Chengdu, P.R. China; 3 Department of Nephrology and Biostatistics Center, West China Hospital, Chengdu, P.R. China

## Abstract

In this study, three-dimensional printing (3Dp) models and simulation surgeries (SSs) were applied in two challenging aortic cases. The first was an abdominal aortic aneurysm with a complex neck, and the second was a thoracic aortic dissection aneurysm (TADA) with an angled arch. In order to avoid unpredictable obstacles and difficulties, we made optimal surgical plans by using 3D models and virtual simulations. Based on preoperative evaluation system, the surgical plans seemed more reasonable and time-saving.

## Introduction

Abdominal aortic aneurysms (AAAs) and thoracic aortic dissection aneurysms (TADAs) are life-threatening entities. Patients may immediately lose their lives after ruptures that primarily result from the combination of the effects of an unstable hemodynamic condition and a weak vessel wall. Estimates of the incidence of AAA vary from 3–117 per 100000 person-years^1^ and 10.4 cases per 100000 for TADA^2^. Due to the mini-invasive characteristics, endovascular interventions (ETs) bring treasurable chance for the weak and advanced age patients^3^. Thanks to the efforts of doctors and engineers over decades, complex anatomic condition is not the absolute forbidden zone of ET any more^4^.

However, some complicated anatomical conditions remain challenging because of indirect visibility of computed tomography (CT), which hardly reflects the exactly precise conditions of complicated cases^5^. In AAA cases with complex neck and TADA with proximal lesions that are extremely close to the left subclavian artery (LSA), selections of the approach, stent graft, and location are difficult, but, as we know, crucial in the hemodynamic condition and prognosis.

Recently, a new evaluation system involving three-dimension printing (3Dp) and simulation surgery (SS) was adopted to overcome these difficulties. In challenging cases, the surgeons were able to have an intuitive view of the anatomical condition and were able to mimic the operation in a 3Dp model. This system helps surgeons in making optimal surgical plans for challenge cases. Here, we share our limited experience of two cases in our institution.

## Clinical Cases

### Case 1

**Figure 1:**
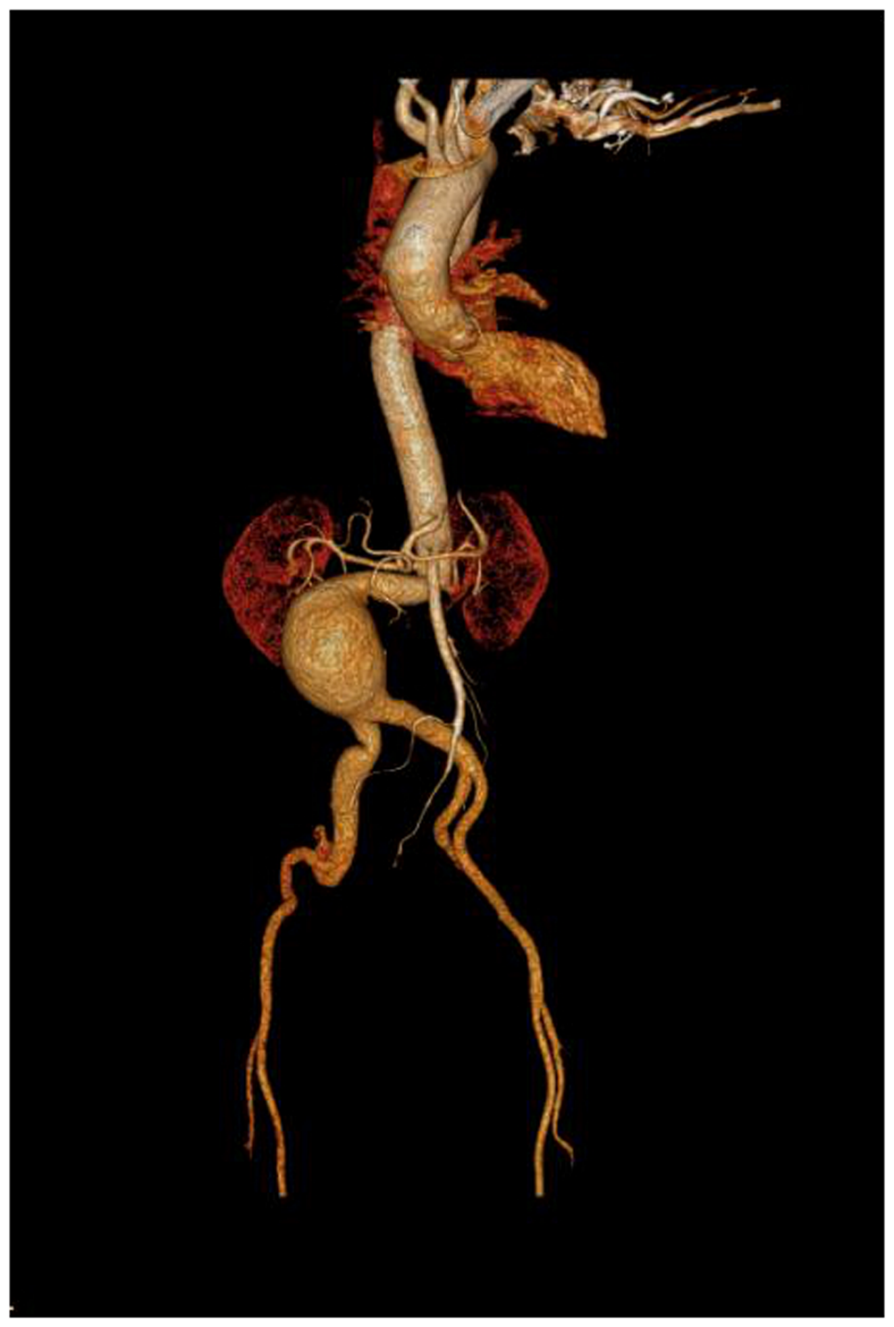
Computed tomography showing an infrarenal AAA with a sharp angled neck and a short neck. The right common iliac artery was severely tortuous.

This case was a 67-year-old female patient. CT revealed an infrarenal AAA with a sharp, angled neck and a short neck. Both angle α and β were nearly 90°, and neck of the aneurysm was 12 mm approximately. The right and left renal arteries (RAs) raised at angle α. The right common iliac artery was 21 mm in length and severe tortuous, and the left was 16 mm, yet without tortuosity (Fig. 1). In this extremely challenging case, it was difficult to predict the changes in shape after the deployment of a stent. It’s known that such changes would directly affect the influx of RA and the adhesion of the struts, and both of them associates with prognosis. Thus, location and approach selection, and intervention after shape change post-deployment were extremely important; however, these factors are far difficult to manage and predict in common methods, e.g., CT in the case.

### Case 2

**Figure 2:**
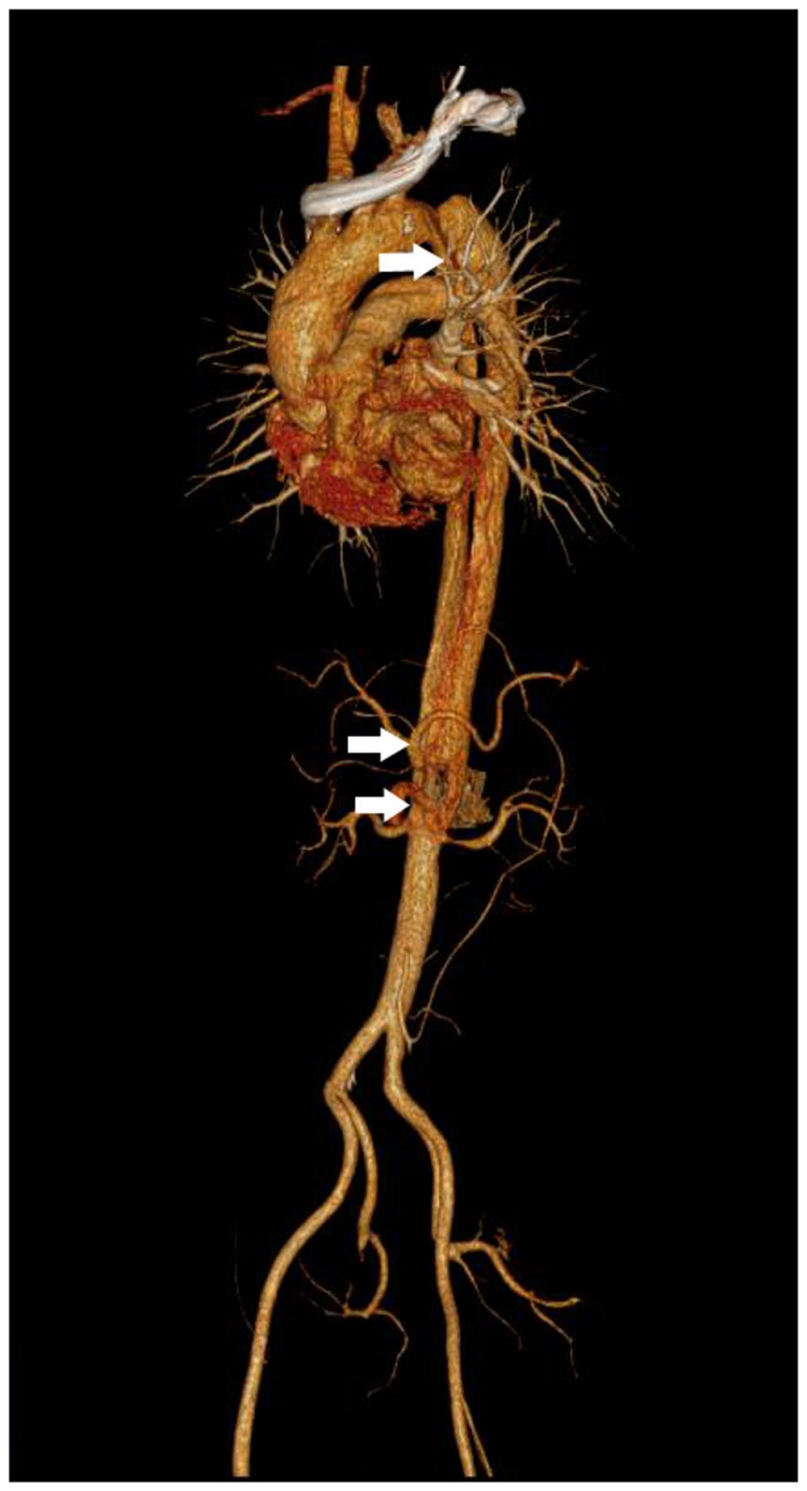
Thoracic aortic dissection aneurysm (Stanford B type) with a type II arch. A proximal lesion was located 30 mm from the site of left subclavian artery. The white arrows indicate lesions.

This case involved a 43-year-old male TADA (Stanford B type) patient. CT revealed type II arch. A proximal lesion located 30 mm away from the left subclavian artery (LSA). The LSA and left carotid artery (LCA) were 9.5 mm and 8.3 mm, respectively. The diameter of aorta at the LSA and LCA segments were 26 mm and 27.5 mm respectively. The dissection involved the coeliac trunk, superior mesenteric artery (SMA) and left RA (Fig. 2), the maximum diameter of true lumen located at the coeliac trunk level with 23 mm. Whether a “chimney” was needed to be adopted ensuring the blood supply of the LSA was the key point of this case. Although the landing zone seemed sufficient, the aortic arch potentially could have become more angled after stent deployment, which would result in new lesion formation or reverse extension.

In mentioned above either AAA or TADA case, it was difficult for the surgeons to decide stent graft and interventional plan. Therefore, we decided to use 3Dp models to investigate the exact anatomic parameters in these cases and to perform simulation surgery to detect the changes in the shapes of the aortas after surgery.

## Materials and Methods

The CT scans were post-processed using standard techniques. The scanning range was from the aortic arch to the synchondroses pubis, and a scanning layer of 0.75 mm was processed via binary CT (SIEMENS, Germany). The data were exported as digital imaging and communications in medicine (DICOM) format. The opening of the SMA to the middle segment of the bilateral external iliac artery in a 0.75-mm slice was imported into the MIMIC 16.0 software to reconstruct a 3D image of the AAA. Three-dimensional data were created in the STL format and subsequently imported into the Magic software to smooth the surface. After this smoothing was applied, an STL file was exported; this format is suitable for a variety of 3D printers. A full-sized AAA model was printed by a printer (Objet 260, STRATSYS) using soft material (Tangoplus) that was primarily comprised of silica gel.

All procedures were performed in accordance with the relevant guidelines and regulations. This study was approved by the ethics committee of West China Hospital, Sichuan University. Informed consent was obtained from the two participants.

### Model evaluation

**Figure 3:**
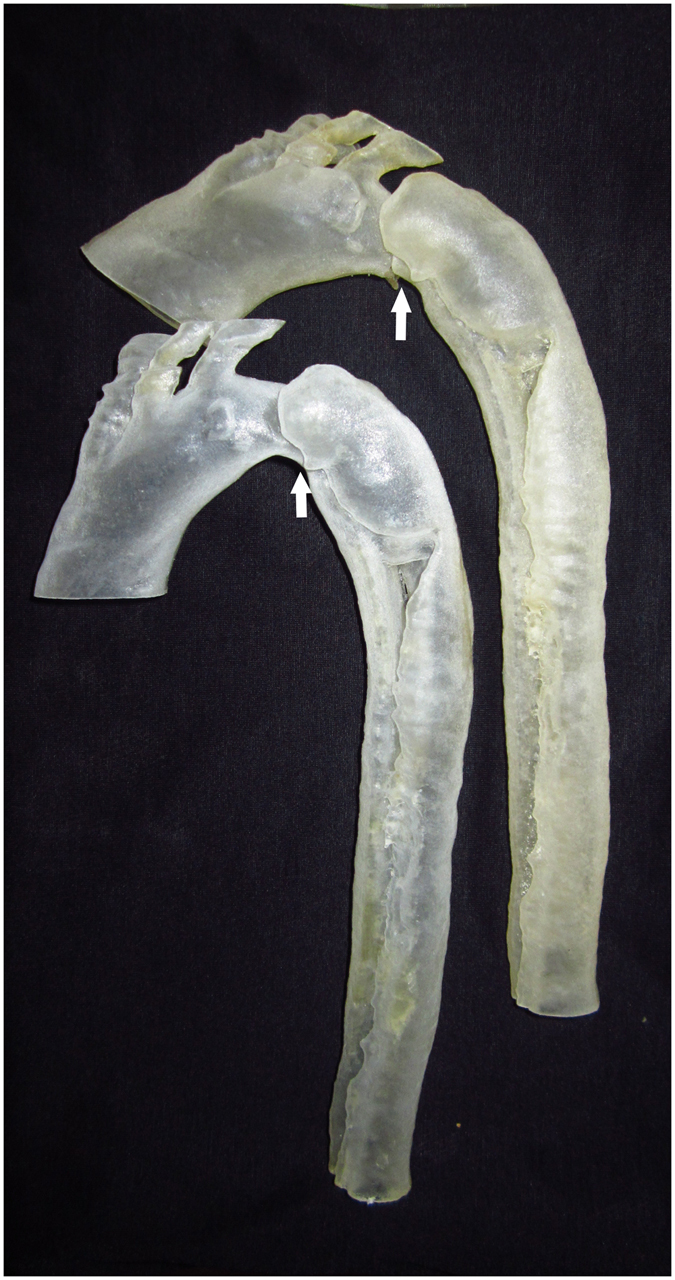
Three-dimensional model of a dissection. The top panel shows the possible shape after stent deployment. The white arrow indicates the maximum angle of the arch.

**Figure 4:**
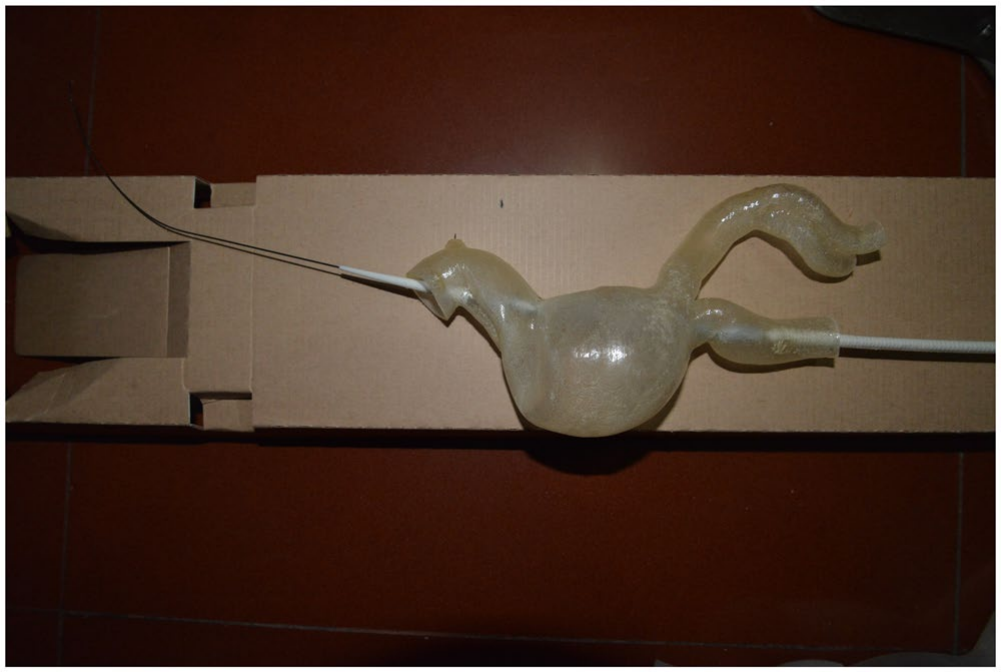
Illustration of a simulation surgery with a stent graft before the intervention.

The 3Dp model was a reliable tool because it provided the surgeons with direct visual representations of the anatomic conditions in AAA and TADA cases. SS, including the wire approach, stent deployment and wire withdrawal was performed in 3Dp model cases. In the TADA 3Dp model and surgery simulation, we needed to confirm the following: (1) the locations of the lesions; (2) the relationships between the LSA, LCA and the proximal lesion; and (3) that the shape change of the arch after deployment was clearly displayed (Fig. 3). In the AAA 3Dp model and SS, we needed to confirm the following: (1) a highly angled neck of the AAA; (2) the relationships between the RA visceral arteries and the neck; (3) shape change of the aorta after implementation; and (4) that the stress-strain relationship between the stent and neck was thoroughly prior to the intervention (Fig. 4).

### Evaluation results

Regarding the AAA, the results from the evaluation system revealed that the right femoral artery was the optimal approach. Moreover, SS proved that release of the main body at the level of the opening of the RA could shorten landing zone of the neck and result in type I endoleak. Therefore, a restrictive stent was needed at the proximal landing zone to prevent endoleak and migration. Moreover, SS also revealed that if the opening of the short limb directed anterolaterally, approach would be smoother.

In terms of TADA, 3Dp revealed that there were 5 lesions on the aorta. Three of these lesions were obvious and located 30 mm away from the end of the LSA and at the level of the coeliac trunk and RA respectively. In the SS, the guidewire was smoothly moved in the true lumen of the TADA and entered into arch. According to our evaluation system, the 1st lesion was 30 mm away from the end of the LSA, and this distance was sufficient for landing. The SS and virtual simulation indicated that landing zone would be much more stressful and could potentially stimulate the formation of a new lesion or reverse the extension of the dissection. Therefore, we selected the “chimney” technique to avoid this potential risk. The diameter of the distal descending aorta was 23 mm according to the CT scan; however, this value was exactly 20 mm according to the measurements in 3Dp model, so use of restrictive stent added into the plan.

### Result of practice

1. When dealing with an AAA in practice, we successfully operated due to the use of the SS. Main body measurements (Medtronic ENBF 2513CC145EE) were smoothly delivered through the right femoral artery through severe tortuosity. Angle of the neck was reduced, and the proximal landing zone shrank. Therefore, a restrictive stent (Medtronic ENBF 2525C45EE) was placed at the neck zone for reinforcement as planned. A short limb (Medtronic ENLE 1616C120EE) was successfully deployed, and an extension stent (Medtronic ENLW 1620C95EE) was deployed via the femoral artery (Fig. 5). A repeated angiograph revealed that the AAA was completely isolated except for a slight I type endoleak.

**Figure 5:**
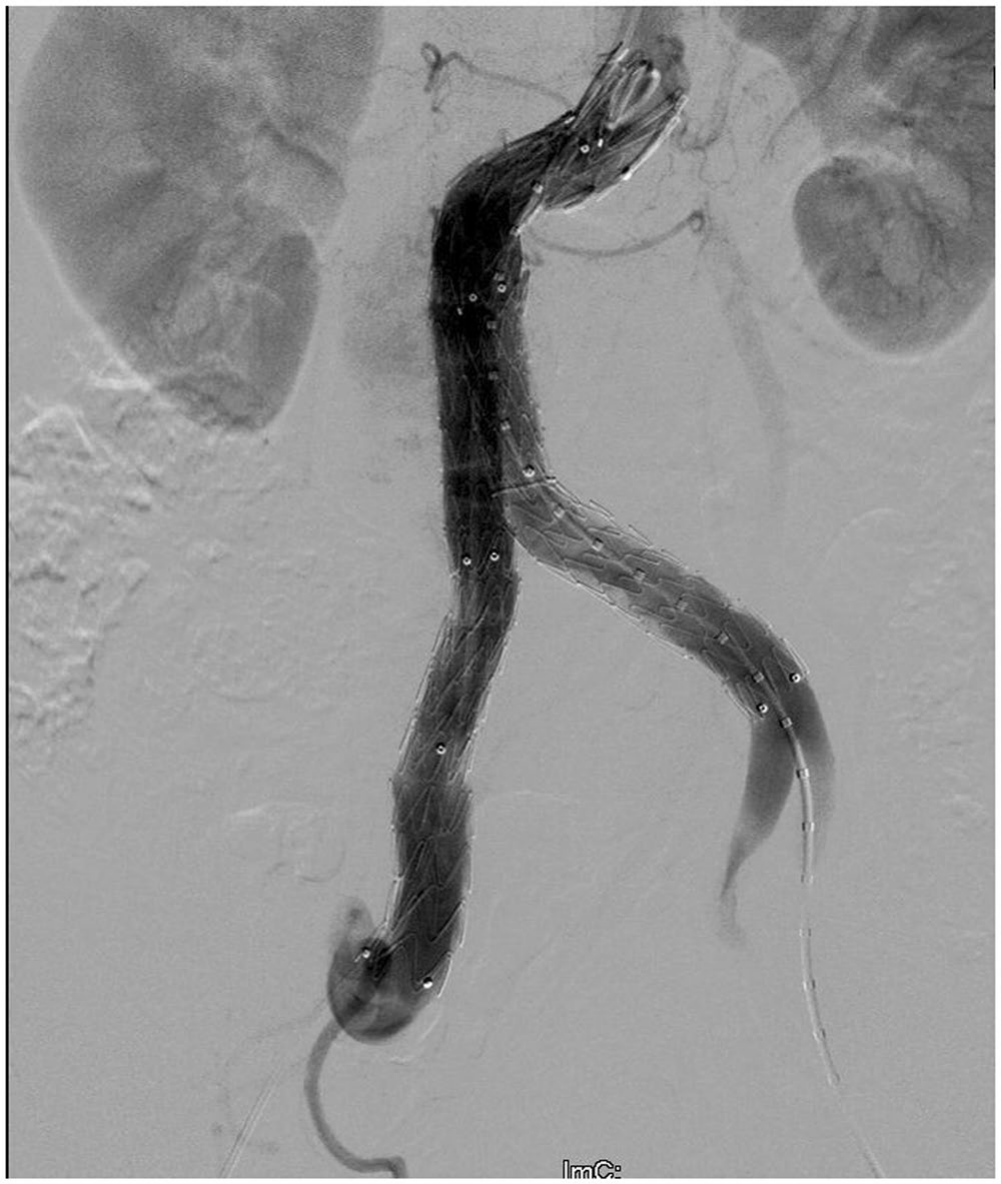
Angiograph after stent graft deployment. The surgical plan (i.e., the choice of approach and stent) was smoothly created based on the preoperative simulation surgery.
2. In the TADA practice, like the SS, the guidewire moved smoothly in the true lumen and entered into the arch through the right femoral artery. A restrictive stent (Medtronic ENDURANT ENEW2020C80EE) was placed at the end of the descending aorta. Next, the covered stent (Medtronic VALIANT VAMF2828C150TE) was deployed from the opening of the LSA into the restrictive stent. The proximal end of an FLUENCY stent (8*60 mm) was inserted into arch, and the distal end was placed before the opening of the vertebral artery (Fig. 6). A repeated angiograph revealed that the LSA and the visceral arteries were very well revealed. The postoperative shape of the arch maintained a good appearance.

**Figure 6:**
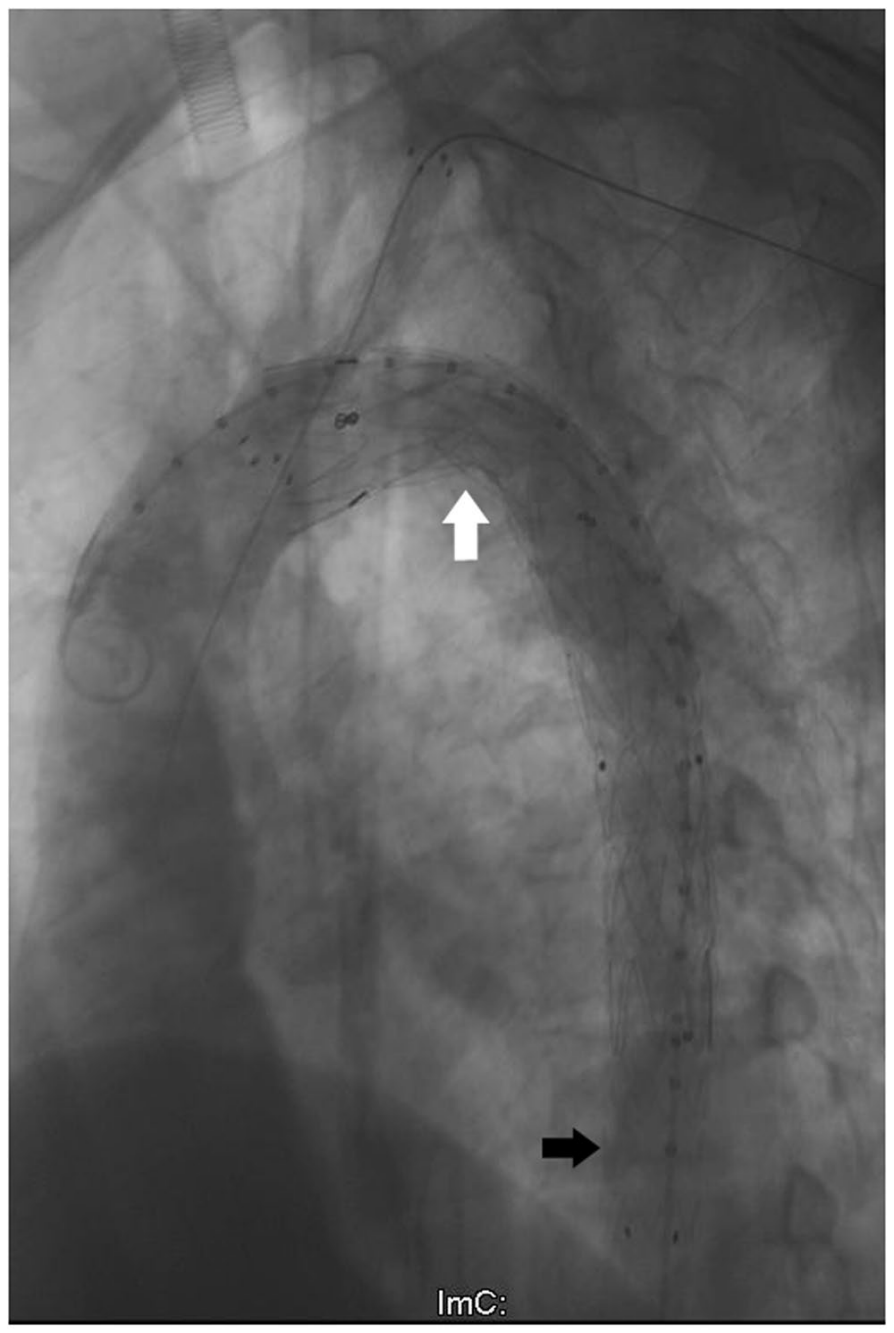
Angiography after stent graft deployment. The shape of arch accorded with the results of the simulation. The white arrow indicates the reshaping of the aortic arch, and the black arrow indicates the distal restrictive stent.

## Discussion

Three-dimensional printing models have been proven to be a useful tool in reconstructive surgery, maxillofacial surgery, neurosurgery, orthopedics, and cardiovascular surgery^6–8^. In recent years, 3Dp models have gradually been adopted in vascular surgery, especially for cases of AAA and TADA.

Compared with conventional CT scans, 3Dp models enable greater direct visibility of the aneurysm. In challenging cases of AAA and TADA, both CT scans and CT three-dimensional reconstructions minimally present the precise anatomic condition of the aorta^5^. In terms of demonstrating the spatial relations between aorta and the branches and visceral arteries, 3Dp models have an incomparable advantage over conventional auxiliary examinations. In our study, it was easy to reveal that the anatomical parameters were measured more exactly, and as we know, these data are crucial for enabling surgeons to make optimal surgical plans and select suitable stents^9, 10^.

Furthermore, SS can be performed before surgery based on the 3Dp model. SS in a 3Dp model could guide surgeons in the selection of the appropriate approach, which is important in time-saving. Moreover, SS ensures the avoidance of obstacles in the use of the guidewire, especially in the true lumen of TADA. For example, in case 2, although the proximal landing zone was sufficient (30 mm), stress-strain might have resulted in the formation of a new lesion or the reverse extension of the dissection according to the virtual simulation before surgery^11^. Therefore, we decided to adopt the “chimney” technique to reduce the stress-strain at the arch site rather than the direct deployment. This technique may be extremely beneficial for patient’s short and long-term prognosis.

Unfortunately, there are no case-control studies that confirm that this preoperative evaluation system is effective. Currently, we are rebuilding images of all our complex aortic cases according to previous clinical data and analyzing stress conditions to identify the patients whose surgical plans seemed unreasonable given the current mechanical perspective. Moreover, we are comparing the prognoses of these patients with those of other patients. We believe that imminent results will effectively prove that this evaluation system works. Simultaneously, preoperative 3Dp models and SSs become routine evaluation methods for challenging cases, such as the treatment of thoracoabdominal aneurysms with fenestration covered stents, which can be customized according to 3D models and SS before surgery.

In conclusion, this preoperative evaluation system including 3Dp model, SS and virtual simulation could help surgeons to decide optimal plans, especially in difficult cases.
